# Left vocal cord paralysis revealing chronic mediastino-pulmonary silicosis: a case report

**DOI:** 10.11604/pamj.2025.51.106.48514

**Published:** 2025-08-26

**Authors:** Abdellatif Outrah, Amine Hafidi, Youssef Bouktib, Ayoub El Hajjami, Badr Boutakioute, Mariem Ouali Idrissi, Najat Cherif Idrissi El Ganouni

**Affiliations:** 1 Service de Radiologie, Hôpital Arrazi CHU Mohamed VI, Marrakech, Morocco

**Keywords:** Silicosis, vocal cord paralysis, calcified mediastinal adenopathy, occupational lung disease, case report

## Abstract

Silicosis is one of the most common occupational lung diseases, resulting from prolonged inhalation of crystalline silica dust. We report the case of a 66-year-old man with a history of silica exposure who presented with hoarseness due to left vocal cord paralysis. Imaging revealed calcified mediastinal lymphadenopathy compressing the left recurrent laryngeal nerve, leading to the diagnosis of chronic mediastino-pulmonary silicosis. This rare presentation highlights the need to consider silicosis as a differential diagnosis in cases of unexplained vocal cord paralysis, especially in individuals with occupational exposure to silica.

## Introduction

Silicosis is a well-known, irreversible occupational lung disease caused by chronic inhalation of respirable crystalline silica particles [1]. It remains a global public health concern, particularly in regions with limited regulatory enforcement on occupational safety [1]. The disease typically affects workers in industries such as mining, quarrying, masonry, and agriculture [1,2]. Clinical presentation varies and may be asymptomatic or present with nonspecific respiratory symptoms such as chronic cough and dyspnea [1]. Advanced stages may lead to significant fibrosis and complications, including pulmonary hypertension, tuberculosis, and lung cancer. One rare but clinically important complication is compressive lymphadenopathy affecting adjacent structures, such as the recurrent laryngeal nerve [2]. This can result in vocal cord paralysis and manifest as dysphonia or hoarseness [3]. We report a case of chronic silicosis revealed by left vocal cord paralysis due to mediastinal lymphadenopathy compressing the left recurrent laryngeal nerve.

## Patient and observation

**Patient information:** a 66-year-old male with a history of chronic cough and exertional dyspnea presented with voice hoarseness that had evolved over the previous four months. He had a 30-year history of smoking and had worked in rural occupations involving high exposure to silica dust, including masonry, farming, and well digging. There was no history of tuberculosis, surgery, trauma, or thyroid disease.

**Clinical findings:** on physical examination, the patient was hemodynamically stable. No lymphadenopathy, hepatosplenomegaly, or skin abnormalities were noted. The ear-nose-throat (ENT) examination revealed left vocal cord paralysis on laryngoscopy without intrinsic laryngeal lesions.

**Figure 1:**
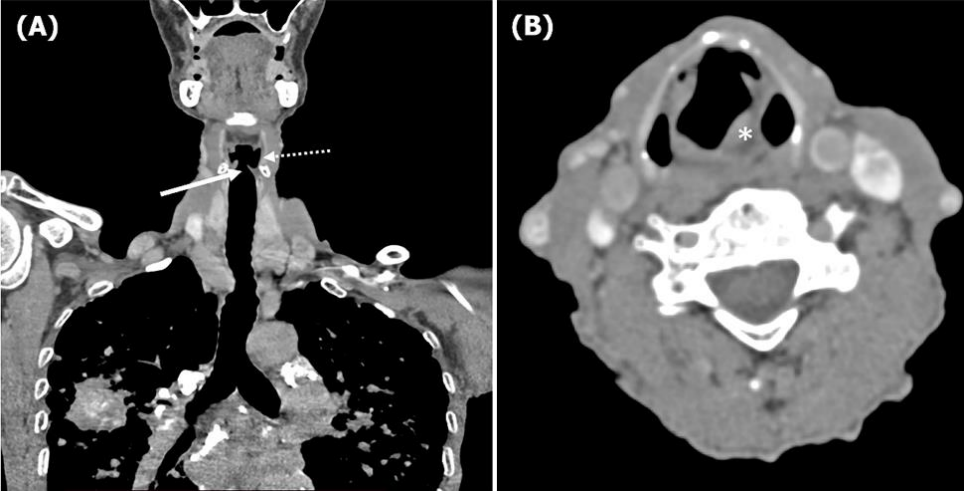
chest computed tomography scan in mediastinal window on coronal (A) and axial (B) views revealing signs of left vocal cord paralysis, most notably medialization and thickening of the aryepiglottic fold (asterisk), along with enlargement of the laryngeal ventricle “sail sign” (dotted and solid arrows)

**Figure 2:**
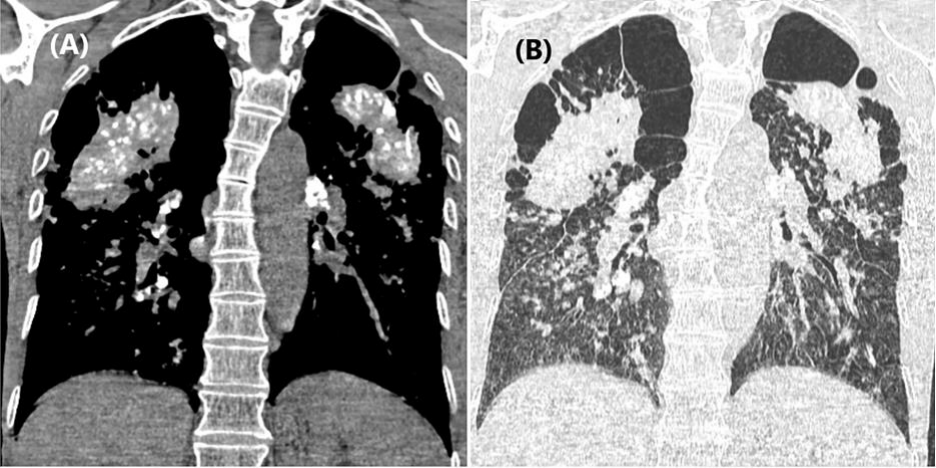
chest computed tomography scan in mediastinal (A) and parenchymal (B) windows on coronal view, revealing large bilateral peri-hilar fibrotic masses involving the upper lobes with massive bullae of emphysema predominant in the apices

**Figure 3:**
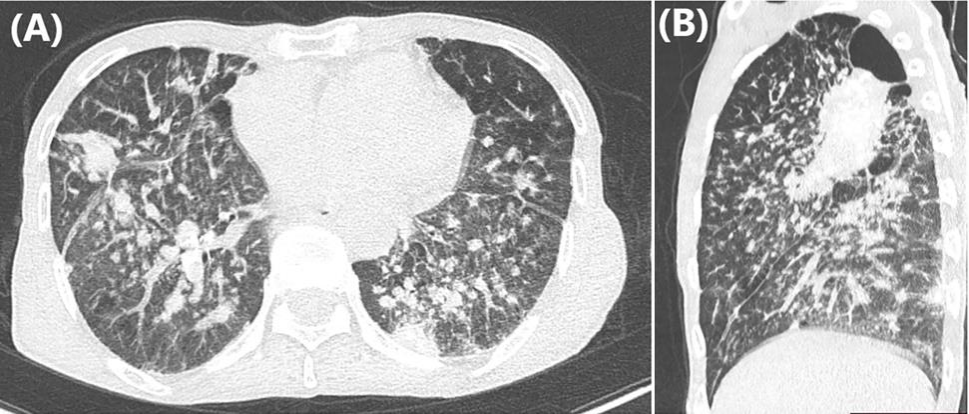
chest computed tomography scan in parenchymal window on axial (A) and sagittal (B) views showing multiple bilateral peri-lymphatic parenchymal and subpleural nodules with pseudo-plaques predominant in the upper lobes

**Figure 4:**
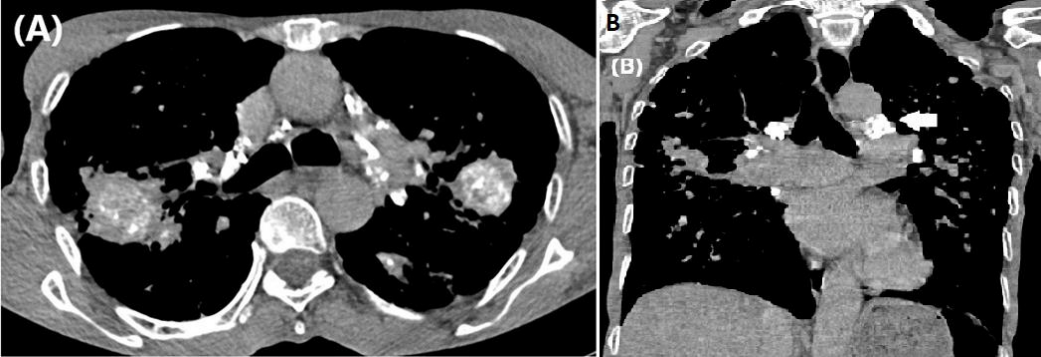
chest computed tomography scan in mediastinal window on axial (A) and coronal (B) views showing the presence of multiple lymphadenopathies involving all territories, most notably the aortopulmonary window (arrow), with peripheral calcifications forming an “egg-shell“ pattern

**Diagnostic assessment:** blood work was unremarkable. A chest computed tomography (CT) scan revealed signs of left vocal cord paralysis, most notably medialization and thickening of the aryepiglottic fold, along with enlargement of the laryngeal ventricle “sail sign” (Figure 1). It also showed large bilateral perihilar fibrotic masses involving the upper lobes, with massive bullae of emphysema predominantly located in the apices (Figure 2). These findings were associated with multiple bilateral peri-lymphatic parenchymal and subpleural nodules, as well as pseudo-plaques, predominantly in the upper lobes (Figure 3). On mediastinal window settings, numerous lymphadenopathies were observed across all mediastinal territories, particularly in the aortopulmonary window. These lymph nodes exhibited peripheral calcifications forming an “egg-shell” pattern (Figure 4), confirming the diagnosis of chronic, complex mediastino-pulmonary silicosis. The compressive, calcified mediastinal lymphadenopathy was identified as the most probable cause of the patient´s left vocal cord paralysis.

**Diagnosis:** chronic mediastino-pulmonary silicosis complicated by left vocal cord paralysis due to left recurrent laryngeal nerve compression.

**Therapeutic interventions:** the patient was referred for multidisciplinary management. No specific surgical intervention was performed, but follow-up and symptomatic treatment were initiated. Video-mediastinoscopy was considered for nerve decompression, pending pulmonary reassessment.

**Follow-up and outcome of interventions:** the patient remained clinically stable, with persistent hoarseness. Surveillance with imaging and spirometry was scheduled. Annual screening for tuberculosis was also planned due to the increased risk of silicosis.

**Patient perspective:** the patient expressed concern about the irreversible nature of the condition but appreciated the diagnostic clarity and planned follow-up.

**Informed consent:** written informed consent was obtained from the patient for publication of this case.

## Discussion

Silicosis is one of the oldest and most common occupational diseases and constitutes a significant social and economic burden due to its severity and the multitude of complications it can cause, some of which may be fatal [2]. This incurable, debilitating, and sometimes deadly yet preventable disease results from the inhalation of respirable crystalline silica dust, which is released during activities involving rock or sand manipulation (e.g. mining, masonry, farming) [2].

Under normal circumstances, the respiratory system employs several defense mechanisms to prevent the onset of silicosis, notably the mucociliary escalator and the lymphatic drainage system, which help clear silica particles from the airways [1]. However, prolonged exposure or dysfunction of these systems, as seen in smokers, renders them ineffective, leading to the accumulation of silica in the lungs and subsequent disease development [1].

Silicosis is typically categorized into two main forms based on the duration and intensity of exposure [2]. Acute silicosis (or silicoproteinosis) occurs in the setting of high-intensity exposure, with symptoms developing within weeks to five years [1]. In contrast, chronic silicosis arises after at least 10 years of exposure and is characterized radiologically by pulmonary nodules and fibrotic masses. The diagnosis is based on a history of silica exposure, characteristic radiological findings, and exclusion of alternative diagnoses that can mimic silicosis, such as sarcoidosis, tuberculosis, or malignancy [1]. In uncertain cases, confirmation may require mediastinoscopy with lymph node biopsy.

**Figure 5:**
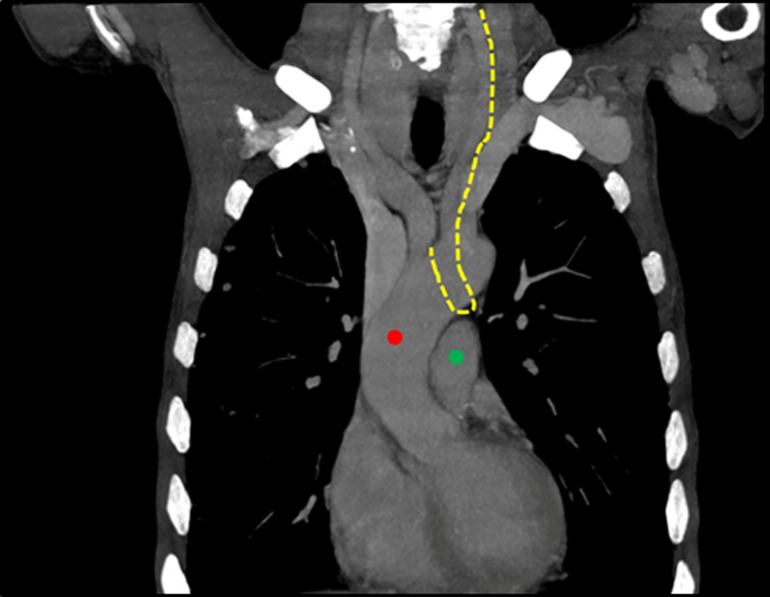
normal chest computed tomography scan showing the presumed trajectory of the left recurrent laryngeal nerve (yellow ling) as it passes through the aortopulmonary window between the thoracic aorta (red circle) and the pulmonary artery (green circle)

Unilateral vocal cord paralysis (VCP) may result from various mediastinal pathologies, including neoplastic (e.g. lung carcinoma, lymphoma), inflammatory (e.g. sarcoidosis), infectious (e.g. tuberculosis), or vascular conditions (e.g. aortic arch aneurysm) [4]. It may also be the first sign of an otherwise occult disease or result from traumatic or central nervous system causes (e.g. multiple sclerosis). As part of the diagnostic evaluation, laryngoscopy is routinely performed to confirm VCP and rule out intrinsic laryngeal lesions such as carcinoma [3]. Emphasizing the unilateral nature of the paralysis is crucial because the left recurrent laryngeal nerve is anatomically more susceptible to injury due to its longer intrathoracic course. It loops under the aortic arch at the ligamentum arteriosum before ascending in the tracheoesophageal groove and entering the larynx at the level of the cricothyroid articulation (Figure 5). Along its course, it lies in proximity to key mediastinal structures, including the aorta, pulmonary artery, esophagus, trachea, and lymph nodes [5].

Cases of silicosis causing hoarseness due to left recurrent laryngeal nerve palsy are rare, with only a handful reported in the literature [6]. In some of these cases, diagnosis was confirmed via mediastinoscopy with lymph node biopsy. In our patient, the diagnosis was based on a combination of long-standing occupational silica exposure, suggestive radiological findings, and the exclusion of other plausible differential diagnoses. The presumed mechanism of VCP was compression of the left recurrent laryngeal nerve by enlarged, calcified mediastinal lymph nodes and surrounding fibrotic changes in the aortopulmonary window, where the nerve courses [4].

Although anti-inflammatory treatments targeting the pathophysiological cascade of silicosis have been explored, no effective therapeutic agents or curative interventions are currently available. In this case, video-assisted mediastinoscopy may offer a benefit by surgically identifying and releasing the recurrent laryngeal nerve from fibrotic entrapment [1].

Close follow-up is essential, including pulmonary function monitoring via spirometry, annual tuberculosis screening, and radiological surveillance of fibrotic lesions. These may evolve into cavitary lesions prone to superinfection (e.g. aspergilloma) or undergo malignant transformation [1].

## Conclusion

This case highlights a rare presentation of silicosis revealed by left vocal cord paralysis due to compressive calcified mediastinal lymphadenopathy. Clinicians should consider chronic silicosis as a potential etiology in patients with unexplained vocal cord paralysis and a relevant occupational history.
